# The efficacy of Australian essential oils for the treatment of head lice infestation in children: A randomised controlled trial

**DOI:** 10.1111/ajd.12626

**Published:** 2017-03-07

**Authors:** Kerryn A Greive, Tanya M Barnes

**Affiliations:** ^1^ Ego Pharmaceuticals Braeside Victoria Australia

**Keywords:** eucalyptus oil, head lice, lemon tea tree oil, piperonyl butoxide, pyrethrin, resistance

## Abstract

**Background:**

The increase in resistance of head lice to neurotoxic pediculicides and public concern over their safety has led to an increase in alternative treatments, many of which are poorly researched or even untested.

**Methods:**

A multicentre, randomised, assessor‐blind, parallel‐group trial (Trial 1) was conducted to compare the safety and efficacy of a head lice treatment containing Australian eucalyptus oil and *Leptospermum petersonii* (EO/LP solution; applied thrice with 7‐day intervals between applications) with a neurotoxic treatment containing pyrethrins and piperonyl butoxide (P/PB mousse; applied twice with a 7‐day interval) in children. A single‐blind, open trial (Trial 2) was conducted to assess the efficacy of EO/LP solution following a single application. In addition, skin irritancy and sensitisation tests using EO/LP solution were performed in adults and children*. In vitro* tests were performed to further assess the ovicidal and pediculicidal efficacy of EO/LP solution.

**Results:**

EO/LP solution was found to be more than twice as effective in curing head lice infestation as P/PB mousse in per‐protocol participants (Trial 1; 83% *vs* 36%, *P *< 0.0001), and was also found to be 100% pediculicidal following a single application (Trial 2). Adverse events were limited to transient itching, burning or stinging. Further skin testing with the EO/LP solution reported no irritation or sensitisation in adults, or irritation in children. *In vitro* exposure of lice and eggs to the EO/LP solution resulted in 100% mortality.

**Conclusion:**

The efficacy, safety and relative ease of use of the EO/LP solution make it a viable alternative in treating head lice.

**Abbreviations**
EO/LP: eucalyptus oil and *Leptospermum petersonii*
ITT: intention‐to‐treat
P/PB: pyrethrins and piperonyl butoxide
PP: per‐protocol

## Introduction

Infestation with head lice is one of the most common parasitic infestations of humans worldwide.^1^ Schoolchildren aged between 3 and 14 years are generally the most affected group, with the prevalence of lice infestation estimated to be between 6–12 million children in the USA annually.^2^ The main symptoms associated with infestation include itching and discomfort, which may lead to substantial social distress, parental anxiety, embarrassment, and unnecessary absence from school and work.^3^ The cost of head louse infestation in the USA has been estimated to be $1 billion annually.^4^

Traditional head lice treatments include a wide variety of neurotoxins including organochlorines (lindane), organophosphates (malathion), carbamates (carbaryl), pyrethrins and pyrethroids (permethrin, D‐phenothrin).^1^ However, this arsenal of pediculicides has failed to obtain adequate control.^5^ Moreover, their repeated use, residual nature and misapplication has led to the selection of resistant populations of lice in Australia^6^ and worldwide.^7^, ^8^, ^9^, ^10^, ^11^

The increasingly poor performance of neurotoxic head lice treatments and the growing public concern over their use has led to an increase in the commercialisation of alternative treatments.^12^ Plant essential oils and their constituents provide a rich source of bioactive chemicals that are easily extractable and biodegradable and generally have low mammalian toxicity.^13^, ^14^, ^15^ However, very few *in vitro* and clinical studies have evaluated the effectiveness of these alternative compounds, and the bulk remain to be scientifically tested.^16^

The aim of this study was to compare the safety and efficacy of a head lice treatment containing Australian eucalyptus oil and *Leptospermum petersonii* (EO/LP) with a neurotoxic treatment containing pyrethrins and piperonyl butoxide (P/PB) in children (Trial 1). The efficacy of the EO/LP solution in killing head lice after a single application was also examined (Trial 2). To rigorously assess the safety of the EO/LP solution, additional skin irritancy and sensitisation tests were performed in both adults and children. The ovicidal and pediculicidal efficacy of EO/LP solution was also tested *in vitro* to eliminate the confounding effect of reinfestation.

## Methods

### Ethics statement

All trial activities were approved by the Human Research Ethics Committee of the University of Queensland. The study was entered in the Australian/New Zealand Clinical Trial Registry: registration no. NCT00381082.

### Participants

Eligible participants were male and female Queensland primary school children (up to Year 7) with live head lice (adults or nymphs), not just eggs, on the hair or scalp, as determined by visual inspection and dry‐combing of hair with a head lice comb. The combing stopped immediately when live lice were detected. Further inclusion criteria were: participants were available for the trial duration; were willing to abstain from using any other lice treatments, including head lice combs, for the duration of the trial; written informed consent given by their parent or guardian. The exclusion criteria were: a history of allergies to head lice products or the specific components being tested; treatment for lice in the 4 weeks prior to commencing the trial; and the presence of scalp disease. Further, if a participant had primary school‐aged siblings, those siblings were screened and enrolled in the trial if they had head lice.

### Interventions

MOOV Head Lice Solution (Ego Pharmaceuticals, Braeside, Victoria, Australia) containing 11% w/w eucalyptus oil and 1% w/w *Leptospermum petersonii* (EO/LP) and Banlice Mousse (Pfizer Consumer Healthcare Group, West Ryde, New South Wales, Australia) containing 1.65 mg/g pyrethrins and 16.5 mg/g piperonyl butoxide (P/PB). The P/PB mousse was chosen as the neurotoxic comparator for this study as it is a market leader in Australia.

### Trial 1: Efficacy and safety of the EO/LP solution compared to the P/PB mousse

A schematic representation of the trial design is shown in Table 1. After informed consent was obtained, participants meeting the inclusion criteria were identified, randomised and assigned to receive one of the two lice treatments.

**Table 1:** Schematic representation of Trial 1 designed to compare the safety and efficacy of the EO/LP solution to the P/PB mousse in a multicentre, randomized, assessor‐blind, parallel‐group trial

	Day 0	Day 1	Day 7	Day 14	Day 21
Informed consent	All	–	–	–	–
Hair/scalp examination by dry‐combing (combing stopped once live head lice observed)	All	All	–	–	–
Inclusion/exclusion criteria	All	–	–	–	–
Randomisation	All	–	–	–	–
Treatment with EO/LP solution or P/PB mousse	All		All	EO/LP solution group ONLY	–
Hair/scalp examination by wet‐combing (combing stopped once live head lice observed)	–	–	–	P/PB mousse group ONLY	EO/LP solution group only
Adverse events	All	All	All	All subjects	EO/LP solution group only

EO/LP, eucalyptus oil and *Leptospermum petersonii*; P/PB, pyrethrins and piperonyl butoxide.

The EO/LP solution was applied thrice with a week between applications, as per the manufacturers’ instructions, i.e. at day 0, day 7 and day 14. As head lice eggs can take up to 10 days to hatch, the use of three applications was designed to ensure that any lice hatching from eggs laid immediately before the first application would be killed by the third application. Although the P/PB mousse claimed that only one treatment was required to effect a cure, it was applied twice using the manufacturers’ instructions, with a week between applications, i.e. at day 0 and day 7, which is in accordance with the recommendations of the Therapeutic Goods Administration of Australia.^17^

Given the physical differences and differences in smell between treatments, the technicians applying the treatments could not be blinded, but effective blinding of the assessment technicians was achieved by the physical separation of the application and assessment staff and by document control procedures. Participants and parents were also prevented from sighting the treatment used.

During the trial participants were free to wash their hair with ordinary shampoo and conditioner and to comb with standard combs. Participants were free to withdraw from the trial at any time.

### Per‐protocol (PP) assessment

The intention‐to‐treat (ITT) population was defined as all participants post‐randomisation and before treatment commenced. The ITT population was the primary population used for the determination of safety and efficacy. To be considered PP, the participant must have received three treatments of the EO/LP solution or two treatments of the P/PB mousse, each 7 days apart.

In addition, the hair of siblings in grades 1–7 of the enrolled participants was examined. If found to be infected with head lice, this sibling was treated with the same head lice product as the enrolled participant on day 0 and was enrolled in the trial. If the sibling was found not to have live head lice but showed evidence of recent infestation (live or dead eggs), the sibling was wet‐combed out. If the sibling had no live lice, live or dead eggs, no further treatment was undertaken.

### Efficacy assessment

The primary efficacy end‐point was the cure rate 7 days after the last application, i.e. day 14 for the P/PB mousse and day 21 for the EO/LP solution. A cure is defined as the absence of live lice, adults or nymphs, as diagnosed by wet‐combing. Wet‐combing is considered the best technique for diagnosing head lice infestation.^18^, ^19^ For this study the wet‐comb technique was standardised and carried out by trained technicians. The secondary efficacy end‐point was the cure rate at day 1 for both products, defined as the complete absence of live lice as diagnosed by visual inspection and dry‐combing.

### Safety assessment

The ITT population was used for the safety evaluation. The incidence and severity of adverse events was recorded at each visit.

### Trial 2: Efficacy after a single application of the EO/LP solution

A schematic representation of the trial design is shown in Table 2. Participants’ characteristics and the inclusion and exclusion criteria were the same as for Trial 1. During the treatment application, the participants’ eyes were covered and they were unaware of the product being applied.

**Table 2:** Schematic representation of Trial 2 designed to assess the efficacy of EO/LP solution following a single application in a single‐blind, open trial

Performed for all subjects at Day 0
Informed consent
Hair/scalp examination by dry‐combing (combing stopped once live head lice observed)
Inclusion/exclusion criteria
Treatment with the EO/LP solution
Hair/scalp wet‐combed until no more head lice observed. Head lice collected
Head lice observed 30 min post collection and assessed as alive, moribund or dead

EO/LP, eucalyptus oil and *Leptospermum petersonii*.

The EO/LP solution was applied once according to the manufacturer's instructions. Hair was washed, partly dried and non‐medicated hair conditioner massaged through, particularly near the scalp. The conditioned hair was divided into sections and wet‐combed. Each section of hair was combed six times or until no more lice were detected. Each combing was collected on a tissue. If lice were present they were visually inspected for any movement, blotted to remove conditioner, and the essential oils were allowed to evaporate.

The head lice were left to recover and carefully assessed using a method adapted from Speare^15^ 30 min after combing was complete using a magnifying lens and probe as being either alive: able to move and right itself when rolled onto its back; moribund: cannot right itself when rolled onto its back; or dead: no movement of any kind. This time point was chosen since preliminary studies of up to 24 h revealed that if lice were moribund after 20 min, they were significantly incapacitated and death was imminent. The primary efficacy end‐point was the number of children from whom head lice showed no signs of life (neither alive nor moribund) 30 min after combing out.

### Skin irritancy and sensitisation study

The safety of the EO/LP solution was patch tested as previously described.^20^ Briefly, the EO/LP solution (0.2 mL) was dispensed onto a patch which was affixed directly to the skin of the back and left in place for 24 h. This procedure was repeated every Monday, Wednesday and Friday for 3 consecutive weeks, for a total of nine consecutive 24‐h exposures. In the event of an adverse reaction, the area of erythema and oedema was measured prior to the next patch application.

Participants were given a 10 to 14‐day rest period after which a single challenge or retest dose was applied to a previously unexposed test site, which was assessed 24 and 48 h after application.

### Paediatric testing

All participants were examined (scalp, face and neck) on day 1 of the single‐blind study by a paediatrician and enrolled if they met the entry criteria. Inclusion criteria were: pre‐pubescent children; written informed consent given by their parent or guardian. Exclusion criteria were: taking medication likely to interfere with the test, including steroidal and non‐steroidal anti‐inflammatory drugs or antihistamines; enrolled in other tests; known hypersensitivity to soaps, detergents, fragrances or shampoos; dermatological or pre‐existing conditions likely to interfere with the test.

The EO/LP solution was applied by a trained technician according to the manufacturer's instructions at days 0, 7 and 14. At each time point, the participants were evaluated for irritation using the following scale: 0 = no irritation present; + = barely perceptible irritation present; 1 = slight irritation present; 2 = mild irritation present; 3 = moderate irritation present and 4 = severe irritation present. Any additional dermatological finding (rash, skin dryness) to the contact areas (scalp, face and neck) was noted. If any irritation reactions were noted, an additional evaluation of the chest, back and abdomen was performed.

All parents or guardians were instructed to report any adverse experiences or concerns related to the use of the EO/LP solution. Participants were evaluated again 24 h after the last application.

### *In vitro* ovicidal and pediculicidal efficacy

For the *in vitro* tests, human body lice (*Pediculus humanus var humanus*) as opposed to head lice were studied, as they can be kept alive in the laboratory for long periods of time by feeding them on rabbits. This is extraordinarily difficult for head lice, which will feed off humans only. While it is well recognised that there are distinct morphological differences between human head lice and body lice, it has been demonstrated using mitochondrial DNA that they are conspecific, that is, they represent the one species.^21^, ^22^ Therefore, the use of human body lice as a surrogate for human head lice is not unreasonable.

The *in vitro* ovicidal and pediculicidal efficacy of the EO/LP solution was determined as previously described.^20^, ^23^ Briefly, eggs and body lice were exposed to the EO/LP solution for 10 s. Purified water was used as the control. The number of eggs that hatched was recorded after 6, 7, 8, 9 and 10 days. Five replicates of 20 eggs each was performed, giving a total of 100 eggs per group. The results from the replicates were pooled, with the data presented as the total percentage of hatched eggs.

The body lice were observed for 60 min following exposure and classed as alive, moribund or dead according to the definitions adapted from Speare's method^15^ described above. Four replicates of 25 body lice each were performed for the EO/LP solution, giving 100 lice. Two replicates of 25 body lice each were performed for the water control, giving 50 body lice. The results are given as % corrected mortality calculated as follows: corrected mortality = ([% alive in water control—% alive in test]/% alive in water control) × 100.

### Statistics

Based on previous studies, the cure rate among children treated with the EO/LP solution and the P/PB mousse was expected to be approximately 70 and 30%, respectively. It was estimated that 29 participants were required in each group in order to show a statistically significant difference between both groups with 80% power and an overall level of significance of *P *= 0.05. Sufficient participants were enrolled to ensure that a minimum of 29 PP participants per treatment arm completed the protocol.

For trial results, χ^2^ tests with a Bonferroni adjustment were used to compare the treatment groups at each time point, day 1 (secondary efficacy end‐point) and day 14 (primary efficacy end‐point for the P/PB)/day 21 (primary efficacy end‐point for the EO/LP). The analysis was performed for the ITT and PP populations. For the *in vitro* studies, statistical analysis was performed using the unpaired Student's *t*‐test at a significance level of 5%.

## Results

### Trial 1: Efficacy and safety

The participants’ characteristics showed no significant differences between treatment groups in relation to sex, school attended, grade, or hair length (data not shown). Of the 97 children in the ITT population, 76 complied with all aspects of the protocol and were considered PP. Of the PP population, 40 received the EO/LP solution and 36 received the P/PB mousse. Reasons for a participant being deemed not PP were: they did not receive the required dose (1 *vs* 0 for the EO/LP solution and P/PB mousse, respectively); they used alternative head lice treatments during the trial (1 *vs* 2); they failed to comply with sibling control criteria (5 *vs* 10); they failed to appear for assessment at day 21 (1 *vs* 0). One participant treated with the EO/LP solution withdrew from the trial due to an adverse event.

Table 3 shows the analysis for PP participants. A significant difference was found in the number of participants cured in the EO/LP group (83%) compared with the P/PB group (36%; *P *< 0.0001) for the primary efficacy end‐point. No statistically significant difference was found between the EO/LP group (60%) and the P/PB group (47%; *P* = 0.2645) for the secondary efficacy end‐point.

**Table 3:** Trial 1: Cure rates for the primary and secondary efficacy end‐points for per‐protocol participants

Treatment	Primary end‐point† *n/n*, %	Secondary end‐point‡ *n/n*, %
EO/LP solution	33/40 (83)	24/40 (60)
P/PB mousse	13/36 (36)	17/36 (47)
*P* value	<0.0001	0.2645

^†^Cure rate 7 days after the last application, i.e. day 14 for P/PB mousse and day 21 for EO/LP solution. ^‡^Cure rate at day 1 for both products as diagnosed by visual inspection and dry‐combing of the hair and scalp. EO/LP, eucalyptus oil and *Leptospermum petersoni;* P/PB mousse, pyrethrins and piperonyl butoxide.

Table 4 shows the analysis for ITT participants. A significant difference was found in the number of participants cured in the EO/LP group (71%) compared with the P/PB group (33%; *P* = 0.0002) for the primary efficacy end‐point. No statistically significant difference was found between the EO/LP group (55%) and the P/PB group (40%; *P* = 0.1259) for the secondary efficacy end‐point.

**Table 4:** Trial 1: Cure rates for the primary and secondary efficacy end‐points for intention‐to‐treat participants

Treatment	Primary end‐point† *n/n*, %	Secondary end‐point‡ *n/n*, %
EO/LP solution	35/49 (71)	27/49 (55)
P/PB mousse	16/48 (33)	19/48 (40)
*P* value	0.0002	0.1259

^†^The cure rate 7 days after the last application, i.e. day 14 for the P/PB mousse and day 21 for the EO/LP solution. ^‡^The cure rate at day 1 for both products as diagnosed by visual inspection and dry‐combing of the hair and scalp. EO/LP, eucalyptus oil and *Leptospermum petersonii*; P/PB, pyrethrins and piperonyl butoxide.

Of the 97 participants who received at least one treatment, 21 adverse events were reported in 13 participants; 18 in the EO/LP group (mainly transient mild to moderate sensations described as either itchiness, stinging or burning lasting no more than 5 min and requiring no treatment) and three in the P/PB group (two events described as stinging and one of a crawling sensation). Overall, both products were well tolerated, which is supported by the fact that only one participant declined a second application (a 5‐year‐old treated with the EO/LP solution).

### Trial 2: Efficacy after a single application

Following a single application of the EO/LP solution to the hair of the 11 enrolled children, 1418 head lice were collected. All lice were observed to be putatively dead as they were wet‐combed out of the hair. All head lice were re‐examined 30 min after combing was finished and were confirmed dead, as shown in Table 5.

**Table 5:** Trial 2: Number of head lice detected by wet‐combing following a single application of the EO/LP solution

Subject	Number of head lice detected by wet‐combing		
	Alive†	Moribund‡	Dead§
1	0	0	10
2	0	0	17
3	0	0	320
4	0	0	663
5	0	0	5
6	0	0	7
7	0	0	16
8	0	0	8
9	0	0	360
10	0	0	12
11	0	0	0
Total	0	0	1418

^†^Alive, able to move and right itself when rolled onto its back; ^‡^moribund, cannot right itself when rolled onto its back; ^§^dead, no movement of any kind. EO/LP, eucalyptus oil and *Leptospermum petersonii*.

### Skin irritancy and sensitisation study

Altogether 56 participants were enrolled and 53 completed the study. There were three male and 53 female participants aged 28 to 74 years. The three participants who did not complete the study withdrew due to reasons not related to the study protocol. No erythema, oedema or adverse reactions of any kind were observed during the course of the study. Therefore, when tested under semi‐occlusive conditions, the EO/LP solution can be considered to be non‐irritating and non‐sensitising to the skin.

### Paediatric testing

Nine boys and 11 girls aged from 6 months to 4 years were enrolled in the study, with five children in each age group; 6–12 months; 12–24 months; 2–3 years and 3–4 years. All children completed the study. No test‐related irritation was observed by the paediatrician and no safety‐related comments were made by any participants or their parents or guardians at any time during the course of the study. Therefore, the EO/LP solution can be considered to be safe for the use on children's hair and scalp.

### *In vitro* ovicidal and pediculicidal efficacy

Table 6 shows that no body louse eggs hatched 10 days following a 10‐s immersion in the EO/LP solution. However, following immersion in water, 24, 76, 92 and 92% body louse eggs hatched after 7, 8, 9 and 10 days, respectively. The EO/LP solution was therefore found to be 100% ovicidal compared with the water control when tested under the conditions described.

**Table 6:** The percentage of hatched louse eggs up to 10 days following a 10‐s immersion in either the EO/LP solution or water (*n *=* *100 eggs each for EO/LP solution and water control)

Treatment	Percentage of hatched eggs				
	Day 6	Day 7	Day 8	Day 9	Day 10
EO/LP solution	0	0	0	0	0
Water control	0	24 ± 7*	76 ± 4*	92 ± 8*	92 ± 8*

*P <* *0.0001. Results presented as mean ± SEM. EO/LP, eucalyptus oil and *Leptospermum petersonii*.

The data in Table 7 show that 60 min following a 10‐min exposure to the EO/LP solution 100% of the body lice were moribund or dead, while all the body lice treated with water were alive. Although a moribund louse is not yet dead, preliminary studies of up to 24 h showed that if a louse is moribund after 20 min, it is significantly incapacitated and death is imminent. The EO/LP solution was therefore found to be 100% pediculicidal compared with the water control (*P *<* *0.0001) when tested under the conditions described.

**Table 7:** Mortality of body lice 60 min following a 10‐min exposure to either the EO/LP solution or water (*n *=* *100 lice for the EO/LP solution, *n = *50 lice for water control)

Treatment	Alive† (%)	Moribund‡ (%)	Dead§ (%)	Corrected mortality¶ (%)
EO/LP solution	0	25.3 ± 2.5*	74.7 ± 2.5*	100*
Water control	100	0	0	0

*P <* *0.0001. Results presented as mean ± SEM. ^†^Alive, able to move and right itself when rolled onto its back; ^‡^moribund, cannot right itself when rolled onto its back; ^§^dead, no movement of any kind; ^¶^corrected mortality (%), ([% alive in water control – % alive in test]/% alive in water control) × 100. EO/LP, eucalyptus oil and *Leptospermum petersonii*.

## Discussion

Recent reviews have indicated the substantial lack of evidence on the efficacy and safety of alternative head lice treatments, with most of the few randomised controlled trials found to be of low validity.^24^, ^25^ Furthermore, a review by the Therapeutic Goods Administration of Australia found that clinical trials conducted on the effectiveness of head lice treatments were complicated by inconsistencies and methodological flaws, including inappropriate definitions of infestation, failure to exclude inappropriate patients, failure to standardise treatments and the failure to choose an appropriate end‐point for efficacy measurements;^17^ conclusions that are also recognised by the wider scientific community.^26^, ^27^

Trial 1 was designed to address these concerns and included strict criteria for study entry, standardised treatment and assessment regimes, sibling treatment, where appropriate, and the use of a primary efficacy end‐point defined as the complete absence of live head lice 1 week after the final treatment.

In Trial 1 the EO/LP solution was shown to be more than twice as effective in curing head lice infestations as the P/PB mousse, as measured by the complete absence of head lice assessed by wet‐combing 1 week after the final treatment. *In vitro* tests confirmed the potent ovicidal and pediculicidal activity of the EO/LP solution.

Overall, the two treatments were well tolerated by participants with few adverse events reported. Adverse events were generally mild and transient, and limited to itching, stinging or burning and no serious or systemic adverse events were reported. Repeat insult patch tests using the EO/LP solution reported no irritation or sensitisation in adults and paediatric testing also reported no irritation, further highlighting the safety profile of the EO/LP solution.

Pyrethrins are neurotoxins derived from chrysanthemum flowers.^28^ They are formulated with piperonyl butoxide to provide a synergistic effect. Pyrethrins modify voltage‐gated sodium channels by keeping the channel open for abnormally long periods, leading to the spastic paralysis and death of head lice.^28^ Pyrethrins were quite effective when introduced in the 1940s; however, the relatively low effectiveness which was observed in Trial 1 reflects the development of resistance in head lice, which has been documented in Australia^6^, ^29^ and worldwide.^7^, ^8^, ^9^, ^10^, ^11^ Resistance has grown rapidly, with susceptibility patterns varying substantially between countries and even between schools.^28^ The low results for the P/PB mousse could also be, in part, due to the rigorous design features of the clinical study, including the hard end‐point definition for cure as measured 1 week after the last treatment application.

The resistance of head lice to neurotoxic treatments is thought to develop when there is an incomplete kill, or when low levels of therapeutic agents remain on the scalp.^17^ The mechanism of action of essential oils is unknown; however, the essential oils used in the EO/LP solution are volatile and do not remain on the scalp after treatment. This property, along with the fact that the EO/LP solution is lethal to all head lice within a 10‐min treatment is unlikely to cause the rapid rise in resistance that has been seen for traditional neurotoxic products.

So what is the cause of the 18% of treatment failures for the EO/LP solution in Trial 1? The most likely reasons could be (i) that some head lice survived any one of the three treatments; (ii) that some eggs survived any one of the three treatments and (iii) re‐infestation. Trial 2 was designed to examine specifically whether some head lice survived any one particular treatment by wet‐combing after a single application of EO/LP solution. Wet‐combing hair with hair conditioner is considered the best technique for diagnosing the presence of head lice.^18^, ^19^ Wet‐combing with hair conditioner is also used by some researchers to obtain a supply of freshly caught head lice for further research. It has been reported that most, if not all lice, removed in this way can recover after capture.^30^

All participants included in Trial 2 had live lice. The number of head lice were not quantified prior to treatment. However, following the application of the EO/LP solution and washout no head lice were recovered using wet‐combing from one participant, and up to 663 head lice recovered from another participant, that is, the initial infestation ranged from light (<10 lice) to heavy (>600 lice). All 1418 head lice recovered from the 11 participants were putatively dead on wet‐combing from the hair and did not recover when the conditioner was blotted from them and they were allowed ample time to revive. This indicates that the EO/LP solution kills 100% of head lice irrespective of the degree of infestation in a single 10‐min treatment. Consequently, the 18% treatment failures associated with Trial 1 may be attributed to the EO/LP solution being incompletely ovicidal on the hairy scalp or re‐infestation of the participant prior to the final assessment. Given that the *in vitro* studies indicate that the EO/LP solution is 100% ovicidal after only a 10‐s immersion, it is more likely that the treatment failures are due to re‐infestation.

In conclusion, the EO/LP solution contains a proprietary combination of essential oils that has been shown to be safe and effective in eliminating head lice in Australia. The small failure rate is possibly due to louse eggs surviving a treatment on the hairy scalp, but it is most likely due to re‐infestation. The fact that the EO/LP solution is both volatile and quickly effective means it is unlikely to cause the development of head lice resistance in the community.
